# Digit ratio (2D:4D) and academic success as measured by achievement in the academic degree “Habilitation”

**DOI:** 10.1371/journal.pone.0212167

**Published:** 2019-02-25

**Authors:** Ozan Yüksel Tektas, Lorenz Kapsner, Miriam Lemmer, Polyxeni Bouna-Pyrrou, Piotr Lewczuk, Bernd Lenz, Johannes Kornhuber

**Affiliations:** Department of Psychiatry and Psychotherapy, Friedrich-Alexander-University Erlangen-Nürnberg (FAU), Erlangen, Germany; University of Vienna, AUSTRIA

## Abstract

**Introduction:**

Prenatal androgen exposure has important organizing effects on brain development and therefore on future behavior. Previous research has shown, that the ratio between index finger (2D) and ring finger (4D) (2D:4D) could function as a marker of prenatal androgen effects, with a relatively shorter 2D indicating a higher prenatal androgen exposure. 2D:4D is associated with status-seeking and competitive behavior but also with altruism. Therefore, 2D:4D should be related to academic success.

**Methods:**

We examined the 2D:4D of both hands, as well as the difference between both variables (Dr-l), of 209 university graduates (74 women) employed at the Medical Faculty of the Friedrich-Alexander-University Erlangen-Nürnberg, Germany, and we assessed the relationship of these variables with academic career performance. Career performance was measured by the number of publications as first or last author, and by achievement of an academic degree used in many European countries, the so-called “Habilitation”.

**Results:**

In a within-sex analysis we found a non-monotonic association between the right hand digit ratio and the probability of having a “Habilitation” in females. Academic success, measured by the number of publications as first or last author and the probability of a "Habilitation", increases with age. In agreement with the literature, we found higher academic success in men.

**Conclusion:**

We found a non-monotonic relationship between right hand 2D:4D and academic success in females. However, the significance of this relationship was weak.

## Introduction

Prenatal sex steroids have an influence on digit development. High prenatal androgen exposure results in a shorter second digit (2D) relative to the fourth digit (4D) in the adult human hand [1,2]. However, 2D:4D may be determined not only by prenatal testosterone, but also additional factors, such as prenatal corticosterone [3] and genetic factors [4]. The 2D:4D ratio among men is usually smaller than among women [2,5,6]. Additionally, prenatal androgen exposure has an organizing effect on the structure and function of the brain [7]. Previous associations of 2D:4D with variables of interest have been shown e.g. in meta-analyses for athletic prowess [8], autism spectrum disorder [9] and sexual orientation in women [10]. We found a lower 2D:4D ratio among alcohol-dependent patients [11,12], young males with video game addiction [13] and male suicide victims [14]. Several studies have reported a linear relationship between 2D:4D and the traits investigated (e.g., [11,13–17]). A low 2D:4D is typically associated with outcomes that mark status (e.g., success in sports, reproduction) or with traits that either lead to, maintain, or reflect high status (e.g., dominance) [18]. It has been hypothesized that persons with low 2D:4D have a higher need for achievement and therefore seek out, in both their hobbies and jobs, specific domains where they have the abilities required to excel [18–21]. However, some studies report a curvilinear relationship (e.g., [18,22–24]), which has been found e.g. for academic [25] and mathematics performance [22], targeting reaction time in adults [26] and children [23] as well as altruism [24,27].

This study aimed to analyze the relationship between 2D:4D and career performance among university graduates at a medical faculty. We used two measures of academic success: Number of publications as first or last author and the achievement of a “Habilitation”. In the medical field, the first and last author positions are the most important and indicate a high level of involvement and effort. The “Habilitation”, often translated into “postdoctorate degree”, is a scholarly convention of many European university systems; it is a required formal qualification to teach at the university level and also, in most cases, a prerequisite for receiving a full professorship at a university. To achieve a “Habilitation,” academics must demonstrate achievements in academic teaching and research; the latter is measured–at minimum–by the number and impact factor of publications in peer-reviewed journals.

Considering the partially contradictory results in the studies mentioned above, we hypothesized that 2D:4D is related to academic success as assessed by the completion of more publications as first or last author and a “Habilitation”. We opine that scientific work and completion of a “Habilitation” is not only driven by strive for status, but instead also is a long-term effort and that successful academics have to be also “team players” and have to show the ability to address setbacks (see discussion). We conducted an exploratory data analysis to investigate possible relationships between 2D:4D and academic success as measured by the number of publications as first or last-author and achievement in the academic degree “Habilitation”.

### Methods

This study was part of the Finger-Length in Psychiatry (FLIP) project of the Department of Psychiatry and Psychotherapy of the University Hospital in Erlangen [11–14,28]. The investigation was conducted according to the principles expressed in the Declaration of Helsinki, and the study was approved by the local ethics committee (Ethik-Kommission der Friedrich-Alexander-Universität Erlangen-Nürnberg, No. 54_14B).

Between April 2014 and November 2015, 210 subjects participated in the study. One participant later withdrew his consent. Participants were recruited by contacting physicians and scientists employed at the clinical and preclinical departments of the Medical Faculty of the Friedrich-Alexander-University Erlangen-Nürnberg (FAU). A search for possible participants was performed using the register on the webpage of the Medical Faculty. Employees who agreed to participate in the study were met with, and written informed consent was obtained after a complete description of the study had been provided. Participants were asked to answer a questionnaire in which the following data were inquired: Date of birth; sex; marital status; number of children; handedness and the completion of a “Habilitation”. Additionally, participants were asked whether they had an intention to accomplish a “Habilitation” or if they had already applied for a “Habilitation” and if they held a full or an associate professorship. Furthermore, the questionnaire inquired about the number of scientific publications as first or last author. In a telephone follow-up carried out in April 2018, participants, who in our first survey answered to have an intention to accomplish a “Habilitation”, were questioned about the current state in this regard. Thus, participants that in 2018 still had an intention to accomplish (n = 6) and participants who had already applied for a “Habilitation” (n = 4) but not yet have finished the procedure were considered as subjects with a “Habilitation” in the statistical analyses in addition to the participants that actually held a “Habilitation”. Experience shows that a “Habilitation” in these situations is indeed very often completed in the following years.

Scanning of the hands and measuring of the finger lengths was performed in accordance with our previous reports [11,13]. Scanning of the participants’ hands, using a Plustek OpticSlim 2600 scanner, was conducted prior to examining or analyzing the abovementioned data. To increase accuracy, small marks were drawn on the basal creases of the participants’ index (2D) and ring (4D) fingers before scanning. The left and right hands were scanned separately with palms down. We used the GNU Image Manipulation Program (GIMP, version 2.8.4; www.gimp.org) to measure the lengths of 2D and 4D from the hand scans. This technique provides good reliability [29]. The total lengths of 2D and 4D were measured from the middle of the basal crease to the fingertip; the length was determined in units of pixels. Both fingers of both hands were measured six times each by three independent raters (ML, OYT, PBP), who were blinded to the participants’ data.

Mean values of the measurements made by the three raters were calculated for each finger. For each individual, ratios of the mean values were calculated for the right hand (R2D:4D) and the left hand (L2D:4D). Furthermore, the Dr-l (= R2D:4D –L2D:4D) was calculated.

Statistical analyses were conducted using IBM SPSS statistics version 21 (SPSS Inc., Chicago, IL, USA) and the R software (Version 3.4.1). The calculation of contingency tables (with statistics) and visualization of the multiple logistic regression model’s terms of interests were performed using the R packages “sjPlot” and “sjstats” [30,31]. McFadden’s Pseudo R² was computed using the R package “pscl” [32]. To compute the likelihood ratio test, the R package “lmtest” [33] was used. To calculate the intraclass correlation coefficient (ICC), the R package “psych” [34] was used.

Reliability among the three raters was calculated for each finger separately, for both the right and left hands, using the ICC (two-way random, absolute agreement) [35–37]. The reliability of the three raters was high for both the right hand (2D: ICC   =  0.9994; 4D: ICC   =  0.9997) and the left hand (2D: ICC  =  0.9996; 4D: ICC  =  0.9997). The reliability of the three raters for the study variables was also high (R2D:4D: ICC = 0.9206; L2D:4D: ICC = 0.9591; Dr-l: ICC = 0.7898).

The relationship of sex differences to categorical variables was assessed by measuring the associations of the variables in the contingency tables.

Before conducting the Student’s two-sample t-test (to analyze the normally distributed variables) or the Wilcoxon rank-sum test with continuity correction (to analyze the non-normally distributed variables), deviation from a normal distribution of the continuous variables was tested by the Shapiro-Wilk test and assumption of homogeneity of variances was tested with the Levene's test with the “median” as the center using the R package “car” [38]. The L2D:4D ratio and R2D:4D ratio did not deviate from a normal distribution. Student’s two-sample t-test assuming equal variances was conducted to compare 2D:4D ratios in female and male participants and participants with and without “Habilitation”. The assumption of normality was violated in the L2D:4D ratio of participants with (W = 0.971, p-value = 0.014) and without “Habilitation” (W = 0.986, p-value = 0.420). Cohen’s d statistic was calculated using the R package “effsize” [39].

Dr-l, age and the number of publications as first or last author did significantly deviate from a normal distribution. The assumption of homogeneity of variance was violated in the number of publications as first or last author in participants with and without “Habilitation” (F-value = 28.244, p-value < 0.001).

To investigate the correlational relation of the continuous digit ratios and Dr-l on the number of publications, a multiple linear regression analysis was performed. Furthermore, the influence of the categorical variables “presence of children” (a binary coded variable [yes/no]) and “marital status” on the number of publications was investigated in a multiple linear regression analysis. Due to the significant deviation from a normal distribution of the dependent variable “number of publications as first or last author”, a rank-based inverse normal (RIN) transformation [40,41] was conducted before building the regression models. All linear regression models were controlled for age (in years). More complex linear regression models were compared to the preceding simpler model using the F-test (e.g. comparison of model 3 with model 2). As a goodness-of-fit statistic, R² was calculated for each model. A multiple logistic regression analysis was conducted to investigate the influence of the independent digit ratio variables and Dr-l on the dependent variable “Habilitation” [yes/no]. All logistic regression models were controlled for age (in years). More complex logistic regression models were compared to the preceding simpler model using the likelihood ratio test (e.g. comparison of model 3 with model 2). As a goodness-of-fit statistic, McFadden’s Pseudo R² was calculated for each model. Due to the sex-differences of 2D:4D digit ratios and the sex-differences of habilitated participants in our cohort (see results), all linear and all logistic regression models were built separately for males and females (“within-sex analysis”). To reveal possible non-monotonic associations, squared 2D:4D was introduced to the linear and the logistic regression models. The analyses of the whole cohort are presented in the S1 File.

## Results

Demographic values and measured variables of all participants are presented in Tables 1 and 2. The cohort consisted of 174 physicians and 35 participants with university degrees in the following disciplines: biology (n = 18), biochemistry (n = 4), physics (n = 2), chemistry (n = 1), medical informatics (n = 1), history (n = 1), psychology (n = 1), psychology/French/Spanish/biology (n = 1), statistics (n = 1), microbiology (n = 1), computer visualistics/biology (n = 1), plant production/ biophysics (n = 1), physics/ biology (n = 1) and chemistry/food chemistry (n = 1). The recruitment strategy deliberately resulted in an overrepresentation of participants with “Habilitation” (117 out of 209) in order to ensure sufficient numbers for statistical analysis; however, the sex ratio of almost 3:1 (m:f) of the participants with a “Habilitation” was preserved by the recruitment strategy.

**Table 1:** Demographic values and measured variables of all participants. Continuous variables.

	Female (n = 74)	Male (n = 135)
L2D:4D: (min/ median/ mean/ max/ sd)	0.905/ 0.974/ 0.975/ 1.072/ ±0.031	0.847/ 0.963/ 0.961/ 1.041/ ±0.029
R2D:4D: (min/ median/ mean/ max/ sd)	0.908/ 0.972/ 0.972/ 1.034/ ±0.028	0.874/ 0.962/ 0.960/ 1.043/ ±0.030
Dr-l: (min/ median/ mean/ max/ sd)	-0.102/ 0.001/ -0.003/ 0.038/ ±0.023	-0.054/ 0.001/ -0.001/ 0.053/ ±0.021
Age: (min/ median/ mean/ max/ sd)	29/ 38/ 39.7/ 64/ ±7.4	26/ 40/ 42.0/ 64/ ±8.5
Number of publications as first/last author: (min/ median/ mean/ max/ sd)	0/ 3/ 9.5/ 100/ ±17.4	0/ 8/ 13.8/ 120/ ±17.6

sd = Standard deviation; min = minimum; max = maximum.

**Table 2:** Demographic values and measured variables of all participants. Categorical variables. Contingency tables.

		Female (n = 74)	Male (n = 135)	p-value
Habilitation		32 (43.2%)	85 (63%)	0.009^A^ (φ: 0.190; x^2^: 6.764)
Professorship	none	65 (87.8%)	97 (71.9%)	0.003^A^ (V: 0.238; x^2^: 11.821)
	full	1 (1.4%)	23 (17%)	
	associate	8 (10.8%)	15 (11.1%)	
Marital status	single	37 (50%)	31 (23%)	<0.001^B^ (V: 0.280; x^2^: 16.379)
	married	36 (48.6%)	98 (72.6%)	
	divorced	1 (1.4%)	6 (4.4%)	
Number of children	0	47 (63.5%)	44 (32.6%)	<0.001^A^ (V: 0.306; x^2^: 19.515)
	1	12 (16.2%)	30 (22.2%)	
	2	10 (13.5%)	41 (30.4%)	
	≥3	5 (6.8%)	20 (14.8%)	
(Any number of) Children		27 (36.5%)	91 (67.4%)	<0.001^A^ (φ: 0.298; x^2^: 17.354)
Handedness	ambi	0	7 (5.2%)	0.130^B^ (V: 0.144; x^2^: 4.265)
	left	6 (8.1%)	13 (9.6%)	
	right	67 (90.5%)	112 (83%)	

^A^: Chi-squared test;

^B^: Fisher’s exact test;

x² = Chi-squared; V = Cramér's V; φ = Phi coefficient.

The contingency tables (see Table 2) show strong associations between sex and having a “Habilitation”, sex and professorship, sex and marital status, sex and the presence of children as well as the number of children.

In the Student’s two-sample t-test, the 2D:4D ratios of female individuals were significantly higher than the ratios of male individuals (Table 3). In the Wilcoxon rank-sum test, the median number of publications as first or last author was significantly higher in male individuals (Table 4).

**Table 3:** Results of student’s two-sample t-test.

Sex	Female (mean [sd])	Male (mean [sd])	p-value (t-test statistic [df])	Cohen‘s d statistic (95%-CI)
N:	74	135		
L2D:4D	0.975 (0.031)	0.961 (0.029)	0.001 (3.26 [207])	-0.463 (-0.751; -0.174)
R2D:4D	0.972 (0.028)	0.960 (0.030)	0.004 (2.881 [207])	-0.426 (-0.714; -0.138)
**Habilitation (Sex = Female)**	**No (mean [sd])**	**Yes (mean [sd])**	**p-value (t-test statistic [df])**	**Cohen‘s d statistic (95%-CI)**
N	42	32		
L2D:4D	0.976 (0.034)	0.973 (0.027)	0.663 (0.438 [72])	0.103 (-0.365; 0.571)
R2D:4D	0.970 (0.031)	0.974 (0.023)	0.544 (-0.609 [72])	-0.143 (-0.611; 0.325)
**Habilitation (Sex = Male)**	**No (mean [sd])**	**Yes (mean [sd])**	**p-value (t-test statistic [df])**	**Cohen‘s d statistic (95%-CI)**
N	50	85		
L2D:4D	0.959 (0.026)	0.962 (0.031)	0.524¹ (-0.640 [133])	0.120 (-0.233; 0.472)
R2D:4D	0.957 (0.029)	0.961 (0.031)	0.410 (-0.827 [133])	0.149 (-0.204; 0.502)

sd = Standard deviation; df = degrees of freedom; CI = confidence interval.

(¹Violation of assumptions of Student’s two-sample t-test due to significant deviation from normal distribution).

**Table 4:** Results of Wilcoxon-rank-sum-test with continuity correction.

**Sex**	**Female (median)**	**Male (median)**	**p-value**
N	74	135	
Dr-l	0.001	0.001	0.857
Age	38	40	0.053
Number of publications as first/last author	3	8	0.001
**Habilitation**	**No (median)**	**Yes (median)**	**p-value**
N	92	117	
Dr-l	-0.001	0.002	0.327
Age	37	42	<0.001
Number of publications as first/last author	1	14	<0.001²

Median values and p-values.

(²Violation of assumptions of Wilcoxon-rank-sum-test due to significant differences in Levene's test for homogeneity of variance).

**Fig 1:**
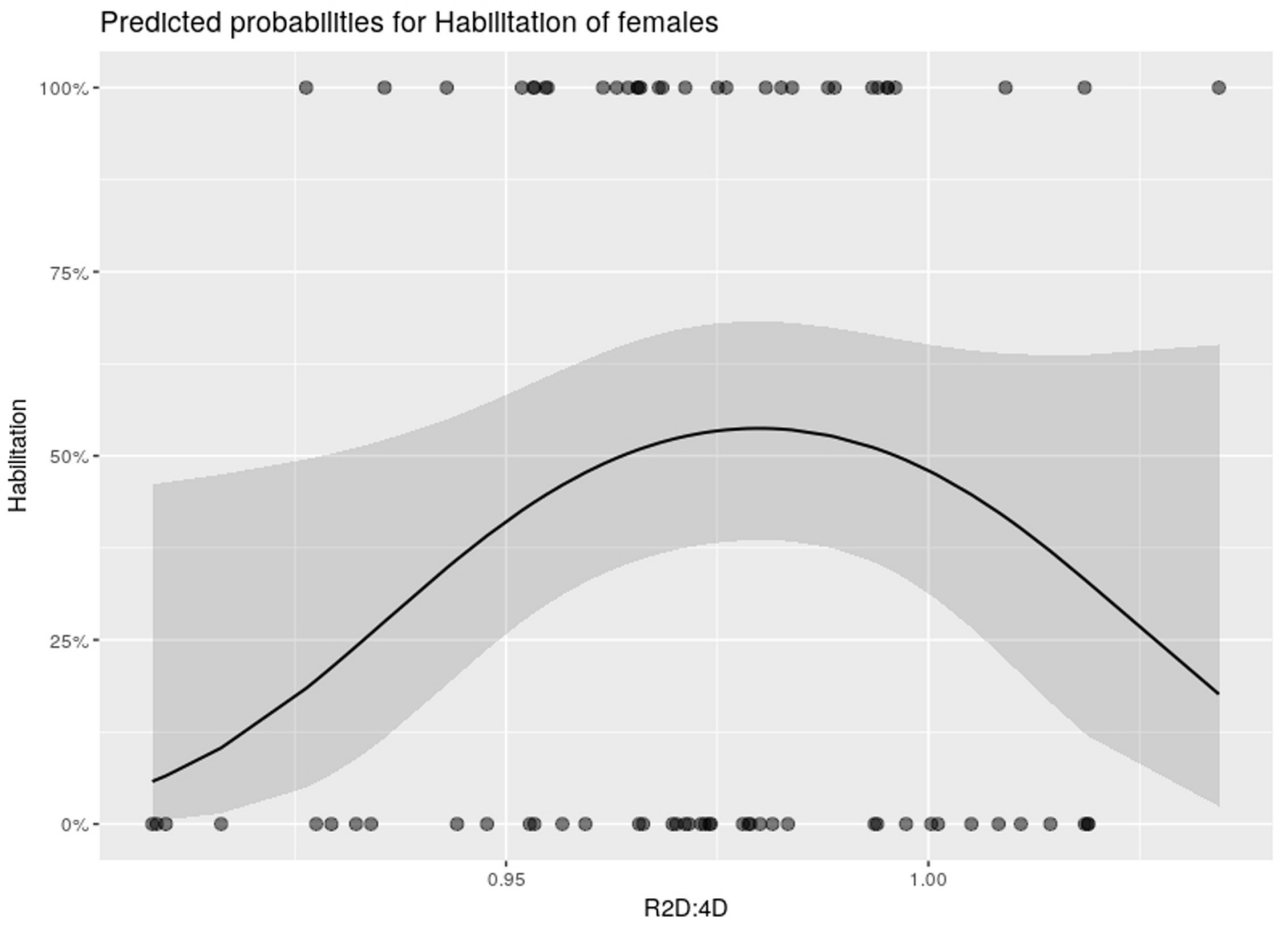
Predicted probabilities for “Habilitation” of females of the model’ term “R2D:4D” from the multiple logistic regression model 3 of Table 5. The plot shows the marginal effects and the raw data points of the model’s digit ratio term of the right hand.

A multiple logistic regression analysis was conducted to investigate the influence of the independent digit ratio variables and Dr-l on the dependent variable “Habilitation” [yes/no]. Table 5 shows the results of the within-sex analysis of the right hand digit ratio. Models 1–3 present the results of the female participants; models 4–6 show the results of the male participants. When introducing the squared 2D:4D digit ratio to the model of females (Table 5, model 3), the likelihood ratio test showed a significant model improvement (p = 0.037) on a significance level α = 5%, compared to the preceding simpler model. In males (Table 5, model 6), this association could not be shown. Fig 1 shows the predicted probabilities for “Habilitation” of females of the digit ratio term of model 3 of Table 5. The within-sex analyses of the left hand digit ratio and Dr-l did not reveal any further significant relationships (Tables 6 and 7).

**Table 5:** Multiple logistic regression: Within-sex analysis of the right hand digit ratio. Models 1–3: females; models 4–6: males. Regression coefficients; p-values in parenthesis.

R2D:4D	*Dependent variable*: *Habilitation*					
Model	(1) Sex = F	(2) Sex = F	(3) Sex = F	(4) Sex = M	(5) Sex = M	(6) Sex = M
Constant	-3.065 (0.026*)	-11.992 (0.192)	-551.044 (0.054)	-2.2 (0.025*)	-6.588 (0.269)	29.466 (0.82)
Age (years)	0.07 (0.04*)	0.076 (0.029*)	0.092 (0.017*)	0.066 (0.005**)	0.065 (0.006**)	0.065 (0.006**)
2D:4D		8.928 (0.324)	1117.532 (0.057)		4.602 (0.455)	-70.681 (0.794)
2D:4D²			-570.215 (0.058)			39.271 (0.781)
**Observations**	74	74	74	135	135	135
**Null deviance (df)**	101.23 (73)	101.23 (73)	101.23 (73)	177.971 (134)	177.971 (134)	177.971 (134)
**Residual deviance (df)**	96.649 (72)	95.647 (71)	91.305 (70)	169.362 (133)	168.799 (132)	168.72 (131)
**Pseudo R² (McFadden)**	0.045	0.055	0.098	0.048	0.052	0.052
**Likelihood ratio test**	LL: -48.324	LL: -47.824/ ChiSq: 1.002/ p: 0.317	LL: -45.653/ ChiSq: 4.342/ p: 0.037*	LL: -84.681	LL: -84.4/ ChiSq: 0.562/ p: 0.453	LL: -84.36/ ChiSq: 0.079/ p: 0.779
**AIC**	100.649	101.647	99.305	173.362	174.799	176.72

Variables: Dependent variable: “Habilitation”; Independent variables: R2D:4D, R2D:4D ², Age (in years).

*p < 0.05;

**p < 0.01;

***p < 0.001;

M = male; LL = Log-likelihoods; x² = likelihood ratio Chi-squared statistic; p = p-value; df = degree of freedom; AIC = Akaike Information Criterion.

**Table 6:** Multiple logistic regression: Within-sex analysis of the left hand digit ratio. Models 1–3: females; models 4–6: males. Regression coefficients; p-values in parenthesis.

L2D:4D	*Dependent variable*: *Habilitation*					
Model	(1) Sex = F	(2) Sex = F	(3) Sex = F	(4) Sex = M	(5) Sex = M	(6) Sex = M
Constant	-3.065 (0.026*)	-3.43 (0.675)	-332.814 (0.154)	-2.2 (0.025*)	-6.734 (0.283)	140.049 (0.333)
Age (years)	0.07 (0.04*)	0.07 (0.044*)	0.079 (0.03*)	0.066 (0.005**)	0.066 (0.005**)	0.066 (0.006**)
2D:4D		0.359 (0.964)	675.556 (0.159)		4.705 (0.463)	-302.137 (0.318)
2D:4D²			-346.056 (0.16)			160.259 (0.31)
**Observations**	74	74	74	135	135	135
**Null deviance (df)**	101.23 (73)	101.23 (73)	101.23 (73)	177.971 (134)	177.971 (134)	177.971 (134)
**Residual deviance (df)**	96.649 (72)	96.647 (71)	94.043 (70)	169.362 (133)	168.822 (132)	167.607 (131)
**Pseudo R² (McFadden)**	0.045	0.045	0.071	0.048	0.051	0.058
**Likelihood ratio test**	LL: -48.324	LL: -48.323/ ChiSq: 0.002/ p: 0.964	LL: -47.022/ ChiSq: 2.604/ p: 0.107	LL: -84.681	LL: -84.411/ ChiSq: 0.54/ p: 0.462	LL: -83.803/ ChiSq: 1.215/ p: 0.27
**AIC**	100.649	102.647	102.043	173.362	174.822	175.607

Variables: Dependent variable: “Habilitation”; Independent variables: L2D:4D, L2D:4D ², Age (in years).

*p < 0.05;

**p < 0.01;

***p < 0.001;

M = male; LL = Log-likelihoods; x² = likelihood ratio Chi-squared statistic; p = p-value; df = degree of freedom; AIC = Akaike Information Criterion.

**Table 7:** Multiple logistic regression: Within-sex analysis of Dr-l. Models 1–3: females; models 4–6: males. Regression coefficients; p-values in parenthesis.Variables: Dependent variable: “Habilitation”; Independent variables: Dr-l, Dr-l², Age (in years).

Dr-l	*Dependent variable*: *Habilitation*					
Model	(1) Sex = F	(2) Sex = F	(3) Sex = F	(4) Sex = M	(5) Sex = M	(6) Sex = M
Constant	-3.065 (0.026*)	-2.873 (0.037*)	-2.438 (0.086)	-2.2 (0.025*)	-2.194 (0.026*)	-2.196 (0.026*)
Age (years)	0.07 (0.04*)	0.066 (0.052)	0.063 (0.067)	0.066 (0.005**)	0.066 (0.006**)	0.069 (0.004**)
Dr-l		13.823 (0.248)	3.335 (0.824)		0.629 (0.944)	-1.654 (0.858)
Dr-l²			-942.273 (0.162)			-269.658 (0.396)
**Observations**	74	74	74	135	135	135
**Null deviance (df)**	101.23 (73)	101.23 (73)	101.23 (73)	177.971 (134)	177.971 (134)	177.971 (134)
**Residual deviance (df)**	96.649 (72)	95.208 (71)	92.65 (70)	169.362 (133)	169.357 (132)	168.634 (131)
**Pseudo R² (McFadden)**	0.045	0.059	0.085	0.048	0.048	0.052
**Likelihood ratio test**	LL: -48.324	LL: -47.604/ ChiSq: 1.441/ p: 0.23	LL: -46.325/ ChiSq: 2.557/ p: 0.11	LL: -84.681	LL: -84.678/ ChiSq: 0.005/ p: 0.944	LL: -84.317/ ChiSq: 0.723/ p: 0.395
**AIC**	100.649	101.208	100.65	173.362	175.357	176.634

*p < 0.05;

**p < 0.01;

***p < 0.001;

M = male; LL = Log-likelihoods; x² = likelihood ratio Chi-squared statistic; p = p-value; df = degree of freedom; AIC = Akaike Information Criterion.

In the multiple logistic regression analysis of the whole cohort, the model containing only age and sex as independent variables, showed a significant positive effect of both variables on “Habilitation” (Table K in S1 File, model 1). We did not find any significant effects of the independent continuous digit ratio variables and Dr-l as well as the categorical variables “presence of children” and “marital status” on the dependent variable “Habilitation”, controlling each model for age and sex (Tables K-M in S1 File, Table O in S1 File, Table Q in S1 File). The within-sex analyses of the categorical variables “presence of children” and “marital status” on the dependent variable “Habilitation” did not reveal any further significant associations (Table N in S1 File, Table P in S1 File).

To investigate the correlational relation of the continuous digit ratios and Dr-l on the number of publications, a multiple linear regression analysis with the RIN-transformed dependent variable “number of publications as first or last author” was conducted. In the analysis of the whole cohort, the model, containing only age and sex as independent variables showed a significant positive effect of both variables on the dependent variable (Table B in S1 File, model 1). The within-sex analysis has not revealed any clear impact of R2D:4D, L2D:4D or Dr-l on the number of publications (Results of linear regression analyses in S1 File).

## Discussion

In this study, we investigated the prenatal testosterone exposure, as assessed by the proxy 2D:4D, in a sample of university graduates in a medical faculty. Similarly, to many previous studies, our study found that the 2D:4D ratios of both hands of female individuals were significantly higher than the ratios of male individuals [2,5,6]. As expected, there were clear associations between sex, age and academic success. Participants with “Habilitation” were significantly older than participants without (Table 4). Male individuals had a higher number of publications as first or last author (Table 4 and results of multiple linear regression analysis [Table B in S1 File, model 1]). When the data from male and female participants are pooled and analyzed together, a false relation can emerge, even when controlling the multiple regression models for sex [42]. Therefore, we conducted a within-sex analysis to investigate our findings for male and female participants separately (Tables 5–7). Both the multiple linear regression and the multiple logistic regression analyses show a strong positive effect of age and male sex on the number of publications as the first or last author as well as on the status of “Habilitation”. The age-effect reflects the fact that scientific success is the result of long-term, continuous work.

We did not find any associations of the independent continuous digit ratio variables and Dr-l as well as the categorical variables “presence of children” and “marital status” on the dependent variable “Habilitation”, when analyzing the whole cohort (supplemental file). However, in a within-sex analysis we found a non-monotonic association between the right hand digit ratio and the probability of having a “Habilitiation” in females. When introducing the squared digit ratio to the multiple logistic regression model of females (Table 5, model 3), a just significant improvement of the model was shown in the likelihood ratio test (p = 0.037) compared to the preceding simpler model. In males (Table 5, model 6), this association could not be shown.

### Scientific work has important peculiarities

Direct competition seems to not be a key factor for scientific success. Rather, scientific knowledge is usually gained in cooperating teams [43]. In contrast to regular training in sports or regular practice of a musical instrument, one can rarely expect continuous success in scientific work. According to the random-impact rule [44], important publications occur at random during the scientific career. Furthermore, publications as first or last author and “Habilitation” status do not result in immediate competitive advantage and leading positions; instead, these lead to professorship and leadership positions only for a minority of scientists and with considerable temporal latency. In the context of a medical faculty and a university hospital, leading positions are more easily attainable as senior physicians, who are associated with more power and higher salaries. This is especially true in the current situation, since in Germany only a small number of physicians are available. These reasons might explain why we did not find higher academic success associated with low 2D:4D. Instead, we found evidence for a non-monotonic relationship between R2D:4D and “Habilitation” in female participants. Although within the majority of literature a linear relationship of 2D:4D to other variables is reported, also non-monotonic relationships of digit ratios have been reported previously. E.g., inverted U-shaped relationships have recently been found between digit ratio and mathematics performance [22], academic performance [25], targeting reaction time in adults [26] and children [23] as well as altruism, where the most generous subjects have intermediate 2D:4D [24,27].

It is worth mentioning that comparable to our results, in the studies of Millet and Dewitte [18], Brañas-Garca et al. [24] as well as Galizzi and Nieboer [27], differences in the 2D:4D ratios were also seen in the right hand. Looking at the literature as a whole, there are mixed results with studies reporting significant associations between 2D:4D and target measures for the right and/or left hand. The reasons for the unstable laterality results are not known.

Similar to women in other high-income countries [45], women in Germany are still underrepresented in higher university positions [46]. An extreme example of this so-called "leaky pipeline" [47] is medicine, in which, although approximately 60% of all students are female, only 10% of the professors are female [48]. We found higher scientific productivity in men (the number of publications as first or last author, “Habilitation”), even when controlling for age and the presence of children. This finding is in agreement with the gender gap identified previously ([49–55]; however, see also [56]). This productivity puzzle may have different explanations: scientific ability, self-selection, social selection, and accumulated disadvantage [57]. It has been discussed whether higher variance in mathematical and other abilities might result in a higher number of males with extremely high abilities [58,59]; otherwise, gender differences in scientific abilities are small [59–61] and therefore probably do not explain the lower number of publications, the lower rate of “Habilitation” and the lower number of leading positions of women. Social selection is also not a valid explanation, as women seem to be *positively* discriminated in job interviews for positions in the STEM field [62,63]; and in the Medical Faculty of Erlangen, the “Habilitation” of women is generously supported financially. However, women seem to be less interested in professional advancement [64,65] and more interested in “people vs. things” [66]. This fits well with the finding that women are often more interested in teaching compared to research [45]. Gedrose et al. [67] also showed using a large population of graduates of German medical faculties that women often have less desire to reach a leadership position and that the traditional family image and the distribution of roles still dominates among young female physicians. The graduation gap in STEM increases with increasing levels of gender equality, as found in western countries [68]. Similarly, gender differences in preferences, such as risk taking, are positively related to economic development and gender equality [69]. Moreover, women's self-confidence in their STEM skills, intelligence and creativity is usually lower than men's self-confidence [70–72]. Taken together, differences in preferences, interests and self-confidence between males and females, and thus self-selection, may explain, at least in part, the higher academic success of men in our study as indicated by the number of first or last authored papers and rate of “Habilitation”.

In our cohort, female participants with intermediate R2D:4D values had a higher probability of achieving a “Habilitation”. However, prenatal testosterone, as assessed by 2D:4D values here, seems to be only one of many factors that may have an influence on scientific success. While the mental abilities of women would, of course, allow for at least a comparably frequent "Habilitation", self-selection seems to discourage many women from pursuing a "Habilitation". As pointed out above, there are women, which seem to be more interested in “people vs. things” than professional advancement and which seem to have lower self-confidence in their STEM skills. This might explain why women in our cohort less often achieved a “Habilitation”, despite the fact that they present more often with intermediate and high 2D:4D values. However, this assumption can only be clarified with new, independent data.

In our sample, there was also a strong association between sex and number of children (Table 2). Presumably, women with children are lost through the “leaky pipeline”. This may also be the reason for the lower proportion of married women compared to men (Table 2). However, in the multivariate analyses, the presence of children did not have a significant effect on either the number of publications as first or last author or on the achievement of a “Habilitation”. These results must be interpreted cautiously because we only analyzed participants who stayed within the “pipeline”.

### Strength of the study

This study is the first to investigate 2D:4D and academic success in a German medical faculty, thus focusing on the achievement of “Habilitation”.

### Limitations of the study

This investigation was performed among physicians and scientists, employed in the clinical and preclinical departments of a medical faculty; it therefore considers academics, who presumably are more often interested in a research career. Physicians and scientists who decided to work in other (non-university) hospitals or in other (also non-medical) fields are not considered. Relatedly, another limitation was the heterogeneity of our study sample consisting of clinically working physicians and theoretically working non-physician scientists. Due to the small number within the subgroups of non-physician scientists, we decided not to statistically analyze differences between the subgroups. Regarding publication behavior, we used only the number of publications as first or last author as a secondary outcome. However, the number of publications does not allow conclusions about their quality. Here, we have limited our analysis to the number of publications because the quality of individual publications is difficult to quantify. The often-used impact factor and similar metrics do not measure the quality of a single publication or the contribution of a single scientist to the publication, but instead measure the average citation frequency of publications in that particular journal. Furthermore, we aimed for a high variance of the dependent variable. As the age increases, the variance in the number of publications is higher than in the citation rate [73]. Our recruitment procedure deliberately resulted in a high number of participants with a “Habilitation” compared to the rate of “Habilitation” among physicians and scientists in general. Our statistical analyses were not corrected for multiple testing, thus the significance of our results must be interpreted with caution. Future studies should investigate the association between 2D:4D ratio and academic success in a less enriched cohort. We used a cross-sectional and correlational design, which does not allow for causal conclusions. In addition, with 2D:4D, we used indirect (rather than direct) quantification of prenatal androgen exposure, which may also be influenced by other factors (see introduction).

### Conclusion

Academic success, measured by the number of publications as first or last author and the probability of a "Habilitation", increases with age and is higher among men. In agreement with the literature, we found higher academic success in men, even when controlling for age and the presence of children. In the within-sex analysis, a “Habilitation” is more likely for females, when there are intermediate 2D:4D values on the right hand; however, the significance of this relationship was only weak.

## Supporting information

S1 Data dictionary (CSV)

S1 R-Script (RMD)

S1 File Table A: Multiple linear regression: within-sex analysis of the right hand digit ratio.
Table B: Multiple linear regression: whole cohort; analysis of the right hand digit ratio.
Table C: Multiple linear regression: within-sex analysis of the left hand digit ratio.
Table D: Multiple linear regression: whole cohort; analysis of the left hand digit ratio.
Table E: Multiple linear regression: within-sex analysis of Dr-l.
Table F: Multiple linear regression: whole cohort; analysis of Dr-l.
Table G: Multiple linear regression: within-sex analysis of the presence of children.
Table H: Multiple linear regression: whole cohort; analysis of the presence of children.
Table I: Multiple linear regression: within-sex analysis of marital status.
Table J: Multiple linear regression: whole cohort; analysis of marital status.
Table K: Multiple logistic regression: whole cohort; analysis of the right hand digit ratio.
Table L: Multiple logistic regression: whole cohort; analysis of the left hand digit ratio.
Table M: Multiple logistic regression: whole cohort; analysis of the Dr-l.
Table N: Multiple logistic regression: within-sex analysis of presence of children.
Table O: Multiple logistic regression: whole cohort; analysis of the presence of children.
Table P: Multiple logistic regression: within-sex analysis of marital status.
Table Q: Multiple logistic regression: whole cohort; analysis of marital status.
(DOCX)
