# Supplementary material for: Digit ratio (2D:4D) and academic success as measured by achievement in the academic degree “Habilitation”
Source: PLoS One. 2019 Feb 25;14(2):e0212167. doi: 10.1371/journal.pone.0212167 (PMC6388918; doi:10.1371/journal.pone.0212167)
Supplement: S1 File — Table A: Multiple linear regression: within-sex analysis of the right hand digit ratio. Table B: Multiple linear regression: whole cohort; analysis of the right hand digit ratio. Table C: Multiple linear regression: within-sex analysis of the left hand digit ratio. Table D: Multiple linear regression: whole cohort; analysis of the left hand digit ratio. Table E: Multiple linear regression: within-sex analysis of Dr-l. Table F: Multiple linear regression: whole cohort; analysis of Dr-l. Table G: Multiple linear regression: within-sex analysis of the presence of children. Table H: Multiple linear regression: whole cohort; analysis of the presence of children. Table I: Multiple linear regression: within-sex analysis of marital status. Table J: Multiple linear regression: whole cohort; analysis of marital status. Table K: Multiple logistic regression: whole cohort; analysis of the right hand digit ratio. Table L: Multiple logistic regression: whole cohort; analysis of the left hand digit ratio. Table M: Multiple logistic regression: whole cohort; analysis of the Dr-l. Table N: Multiple logistic regression: within-sex analysis of presence of children. Table O: Multiple logistic regression: whole cohort; analysis of the presence of children. Table P: Multiple logistic regression: within-sex analysis of marital status. Table Q: Multiple logistic regression: whole cohort; analysis of marital status. (DOCX) [file pone.0212167.s004.docx]

***Results of the linear regression analyses***

When introducing the squared 2D:4D digit ratio to the linear regression model of the left hand in females, a significant effect on the RIN-transformed dependent variable “number of publications” was revealed. This effect also led to an improved model performance as measured by the F-Test (p = 0.034; Table C in S1 File, model 3). In an analysis of the whole cohort, this effect could also be demonstrated for the left hand when introducing the interaction term between the digit ratio variable and gender (Table D in S1 File, model 4). However, the model assumptions in the within-sex analysis were violated as the residuals of Table C in S1 file, models 1 and 2 were not normally distributed, so these results need to be interpreted carefully. Similar effects were almost significant for the right hand (within-sex analysis: Table A in S1 File, model 3; whole cohort: Table B in S1 File, model 4). Furthermore, introducing the squared Dr-l to the linear regression model also led to an almost significant model improvement in females (Table E in S1 File, model 3).

| **R2D:4D** | **Dependent variable: Number of publications** | | | | | |
| --- | --- | --- | --- | --- | --- | --- |
| Model | **(1) Sex = F** | **(2) Sex = F** | **(3) Sex = F** | **(4) Sex = M** | **(5) Sex = M** | **(6) Sex = M** |
| Constant | -1.92 (0.001**) | -8.303 (0.022*) | -171.089 (0.053) | -2.645 (<0.001***) | -2.718 (0.228) | -11.022 (0.814) |
| Age (years) | 0.049 (<0.001***) | 0.053 (<0.001***) | 0.055 (<0.001***) | 0.063 (<0.001***) | 0.063 (<0.001***) | 0.063 (<0.001***) |
| 2D:4D |  | 6.408 (0.072) | 342.554 (0.06) |  | 0.077 (0.974) | 17.403 (0.859) |
| 2D:4D² |  |  | -173.474 (0.065) |  |  | -9.031 (0.859) |
|  |  |  |  |  |  |  |
| **Observations** | 74 | 74 | 74 | 135 | 135 | 135 |
| **R²** | 0.16 | 0.198 | 0.236 | 0.307 | 0.307 | 0.307 |
| **Adjusted R²** | 0.149 | 0.175 | 0.203 | 0.302 | 0.296 | 0.291 |
| **Residual Std. Error** | 0.846 (df = 72) | 0.833 (df = 71) | 0.818 (df = 70) | 0.809 (df = 133) | 0.812 (df = 132) | 0.815 (df = 131) |
| **F-Statistic** | 13.738 (df = 1; 72) | 8.756 (df = 2; 71) | 7.215 (df = 3; 70) | 58.91 (df = 1; 133) | 29.234 (df = 2; 132) | 19.357 (df = 3; 131) |
| **F-Test** | RSS: 51.522 | RSS: 49.215/ F: 3.447/ p: 0.068 | RSS: 46.863/ F: 3.512/ p: 0.065 | RSS: 87.065 | RSS: 87.064/ F: 0.001/ p: 0.974 | RSS: 87.043/ F: 0.031/ p: 0.859 |

Table A: Multiple linear regression: within-sex analysis of the right hand digit ratio. Models 1-3: females; models 4-6: males. Regression coefficients; p-values in parenthesis. Violation of model assumptions: Residuals were not normally distributed in model 1.
Variables: Dependent variable: “Number of publications (RIN-transformed)”; Independent variables: Age (in years), Sex (reference = female), R2D:4D, R2D:4D².
*p < 0.05;**p < 0.01; ***p < 0.001; M = male; RSS = Residual sum of squares; F = F-value; p = p-value; df = degree of freedom

| **R2D:4D** | **Dependent variable: Number of publications** | | | |
| --- | --- | --- | --- | --- |
| Model | **(1)** | **(2)** | **(3)** | **(4)** |
| Constant | -2.535 (<0.001***) | -4.901 (0.01**) | -31.755 (0.427) | -53.002 (0.199) |
| Age (years) | 0.057 (<0.001***) | 0.057 (<0.001***) | 0.058 (<0.001***) | 0.059 (<0.001***) |
| Sex (= M) | 0.316 (0.008**) | 0.345 (0.004**) | 0.346 (0.004**) | 8.146 (0.043*) |
| 2D:4D |  | 2.422 (0.202) | 58.278 (0.483) | 96.764 (0.255) |
| 2D:4D² |  |  | -29.023 (0.501) | -46.166 (0.292) |
| Sex (= M) x 2D:4D |  |  |  | -8.059 (0.053) |
|  |  |  |  |  |
| **Observations** | 209 | 209 | 209 | 209 |
| **R²** | 0.289 | 0.294 | 0.296 | 0.309 |
| **Adjusted R²** | 0.282 | 0.284 | 0.282 | 0.292 |
| **Residual Std. Error** | 0.805 (df = 206) | 0.804 (df = 205) | 0.805 (df = 204) | 0.8 (df = 203) |
| **F-Statistic** | 41.825 (df = 2; 206) | 28.515 (df = 3; 205) | 21.442 (df = 4; 204) | 18.149 (df = 5; 203) |
| **F-Test** | RSS: 133.582 | RSS: 132.525/ F: 1.654/ p: 0.2 | RSS: 132.231/ F: 0.46/ p: 0.499 | RSS: 129.802/ F: 3.798/ p: 0.053 |

Table B: Multiple linear regression: whole cohort; analysis of the right hand digit ratio. Regression coefficients; p-values in parenthesis.
Variables: Dependent variable: “Number of publications (RIN-transformed)”; Independent variables: Age (in years), Sex (reference = female), R2D:4D, R2D:4D².
*p < 0.05;**p < 0.01; ***p < 0.001; M = male; RSS = Residual sum of squares; F = F-value; p = p-value; df = degree of freedom.

| **L2D:4D** | **Dependent variable: Number of publications** | | | | | |
| --- | --- | --- | --- | --- | --- | --- |
| Model | **(1) Sex = F** | **(2) Sex = F** | **(3) Sex = F** | **(4) Sex = M** | **(5) Sex = M** | **(6) Sex = M** |
| Constant | -1.92 (0.001**) | -6.376 (0.059) | -149.269 (0.027*) | -2.645 (<0.001***) | -2.479 (0.293) | -32.484 (0.434) |
| Age (years) | 0.049 (<0.001***) | 0.053 (<0.001***) | 0.057 (<0.001***) | 0.063 (<0.001***) | 0.063 (<0.001***) | 0.064 (<0.001***) |
| 2D:4D |  | 4.401 (0.179) | 296.596 (0.032*) |  | -0.172 (0.943) | 62.613 (0.47) |
| 2D:4D² |  |  | -149.355 (0.034*) |  |  | -32.835 (0.469) |
|  |  |  |  |  |  |  |
| **Observations** | 74 | 74 | 74 | 135 | 135 | 135 |
| **R²** | 0.16 | 0.182 | 0.233 | 0.307 | 0.307 | 0.31 |
| **Adjusted R²** | 0.149 | 0.158 | 0.2 | 0.302 | 0.296 | 0.294 |
| **Residual Std. Error** | 0.846 (df = 72) | 0.841 (df = 71) | 0.82 (df = 70) | 0.809 (df = 133) | 0.812 (df = 132) | 0.814 (df = 131) |
| **F-Statistic** | 13.738 (df = 1; 72) | 7.873 (df = 2; 71) | 7.077 (df = 3; 70) | 58.91 (df = 1; 133) | 29.237 (df = 2; 132) | 19.597 (df = 3; 131) |
| **F-Test** | RSS: 51.522 | RSS: 50.217/ F: 1.941/ p: 0.168 | RSS: 47.076/ F: 4.671/ p: 0.034* | RSS: 87.065 | RSS: 87.061/ F: 0.005/ p: 0.943 | RSS: 86.712/ F: 0.527/ p: 0.469 |

Table C: Multiple linear regression: within-sex analysis of the left hand digit ratio. Models 1-3: females; models 4-6: males. Regression coefficients; p-values in parenthesis. Violation of model assumptions: Residuals were not normally distributed in model 1 and model 2.
Variables: Dependent variable: “Number of publications (RIN-transformed)”; Independent variables: Age (in years), Sex (reference = female), R2D:4D, R2D:4D².
*p < 0.05;**p < 0.01; ***p < 0.001; M = male; RSS = Residual sum of squares; F = F-value; p = p-value; df = degree of freedom

| **L2D:4D** | **Dependent variable: Number of publications** | | | |
| --- | --- | --- | --- | --- |
| Model | **(1)** | **(2)** | **(3)** | **(4)** |
| Constant | -2.535 (<0.001***) | -4.525 (0.017*) | -39.655 (0.216) | -71.92 (0.044*) |
| Age (years) | 0.057 (<0.001***) | 0.058 (<0.001***) | 0.058 (<0.001***) | 0.06 (<0.001***) |
| Sex (= M) | 0.316 (0.008**) | 0.343 (0.005**) | 0.334 (0.006**) | 8.448 (0.037*) |
| 2D:4D |  | 2.011 (0.286) | 74.914 (0.26) | 136.454 (0.061) |
| 2D:4D² |  |  | -37.796 (0.272) | -67.004 (0.072) |
| Sex (= M) x 2D:4D |  |  |  | -8.379 (0.045*) |
|  |  |  |  |  |
| **Observations** | 209 | 209 | 209 | 209 |
| **R²** | 0.289 | 0.293 | 0.297 | 0.311 |
| **Adjusted R²** | 0.282 | 0.282 | 0.283 | 0.294 |
| **Residual Std. Error** | 0.805 (df = 206) | 0.805 (df = 205) | 0.805 (df = 204) | 0.799 (df = 203) |
| **F-Statistic** | 41.825 (df = 2; 206) | 28.285 (df = 3; 205) | 21.539 (df = 4; 204) | 18.301 (df = 5; 203) |
| **F-Test** | RSS: 133.582 | RSS: 132.84/ F: 1.164/ p: 0.282 | RSS: 132.056/ F: 1.229/ p: 0.269 | RSS: 129.467/ F: 4.059/ p: 0.045* |

Table D: Multiple linear regression: whole cohort; analysis of the left hand digit ratio. Regression coefficients; p-values in parenthesis.
Variables: Dependent variable: “Number of publications (RIN-transformed)”; Independent variables: Age (in years), Sex (reference = female), L2D:4D, L2D:4D².
*p < 0.05;**p < 0.01; ***p < 0.001; M = male; RSS = Residual sum of squares; F = F-value; p = p-value; df = degree of freedom.

| **Dr-l** | **Dependent variable: Number of publications** | | | | | |
| --- | --- | --- | --- | --- | --- | --- |
| Model | **(1) Sex = F** | **(2) Sex = F** | **(3) Sex = F** | **(4) Sex = M** | **(5) Sex = M** | **(6) Sex = M** |
| Constant | -1.92 (0.001**) | -1.892 (0.001**) | -1.799 (0.001**) | -2.645 (<0.001***) | -2.64 (<0.001***) | -2.636 (<0.001***) |
| Age (years) | 0.049 (<0.001***) | 0.049 (0.001**) | 0.048 (0.001**) | 0.063 (<0.001***) | 0.063 (<0.001***) | 0.064 (<0.001***) |
| Dr-l |  | 1.737 (0.697) | -4.817 (0.396) |  | 0.501 (0.882) | -0.454 (0.897) |
| Dr-l² |  |  | -183.676 (0.07) |  |  | -119.155 (0.325) |
|  |  |  |  |  |  |  |
| **Observations** | 74 | 74 | 74 | 135 | 135 | 135 |
| **R²** | 0.16 | 0.162 | 0.201 | 0.307 | 0.307 | 0.312 |
| **Adjusted R²** | 0.149 | 0.138 | 0.166 | 0.302 | 0.297 | 0.296 |
| **Residual Std. Error** | 0.846 (df = 72) | 0.851 (df = 71) | 0.837 (df = 70) | 0.809 (df = 133) | 0.812 (df = 132) | 0.812 (df = 131) |
| **F-Statistic** | 13.738 (df = 1; 72) | 6.865 (df = 2; 71) | 5.855 (df = 3; 70) | 58.91 (df = 1; 133) | 29.249 (df = 2; 132) | 19.822 (df = 3; 131) |
| **F-Test** | RSS: 51.522 | RSS: 51.412/ F: 0.158/ p: 0.693 | RSS: 49.047/ F: 3.375/ p: 0.07 | RSS: 87.065 | RSS: 87.05/ F: 0.022/ p: 0.882 | RSS: 86.406/ F: 0.976/ p: 0.325 |

Table E: Multiple linear regression: within-sex analysis of Dr-l. Models 1-3: females; models 4-6: males. Regression coefficients; p-values in parenthesis. Violation of model assumptions: Residuals were not normally distributed in model 1.
Variables: Dependent variable: “Number of publications (RIN-transformed)”; Independent variables: Age (in years), Sex (reference = female), Dr-l, Dr-l².
*p < 0.05;**p < 0.01; ***p < 0.001; M = male; RSS = Residual sum of squares; F = F-value; p = p-value; df = degree of freedom

| **Dr-l** | **Dependent variable: Number of publications** | | | |
| --- | --- | --- | --- | --- |
| Model | **(1)** | **(2)** | **(3)** | **(4)** |
| Constant | -2.535 (<0.001***) | -2.526 (<0.001***) | -2.494 (<0.001***) | -2.497 (<0.001***) |
| Age (years) | 0.057 (<0.001***) | 0.057 (<0.001***) | 0.058 (<0.001***) | 0.058 (<0.001***) |
| Sex (= M) | 0.316 (0.008**) | 0.315 (0.008**) | 0.308 (0.009**) | 0.314 (0.008**) |
| Dr-l |  | 0.711 (0.787) | -1.872 (0.521) | -4.426 (0.374) |
| Dr-l² |  |  | -137.435 (0.049*) | -154.773 (0.039*) |
| Sex (= M) x 2D:4D |  |  |  | 3.639 (0.526) |
|  |  |  |  |  |
| **Observations** | 209 | 209 | 209 | 209 |
| **R²** | 0.289 | 0.289 | 0.303 | 0.304 |
| **Adjusted R²** | 0.282 | 0.279 | 0.289 | 0.287 |
| **Residual Std. Error** | 0.805 (df = 206) | 0.807 (df = 205) | 0.801 (df = 204) | 0.803 (df = 203) |
| **F-Statistic** | 41.825 (df = 2; 206) | 27.782 (df = 3; 205) | 22.118 (df = 4; 204) | 17.724 (df = 5; 203) |
| **F-Test** | RSS: 133.582 | RSS: 133.534/ F: 0.074/ p: 0.786 | RSS: 131.008/ F: 3.922/ p: 0.049* | RSS: 130.748/ F: 0.404/ p: 0.526 |

Table F: Multiple linear regression: whole cohort; analysis of Dr-l. Regression coefficients; p-values in parenthesis.
Variables: Dependent variable: “Number of publications (RIN-transformed)”; Independent variables: Age (in years), Sex (reference = female), Dr-l, Dr-l².
*p < 0.05;**p < 0.01; ***p < 0.001; M = male; RSS = Residual sum of squares; F = F-value; p = p-value; df = degree of freedom.

| **Presence of children** | **Dependent variable: Number of publications** | | | |
| --- | --- | --- | --- | --- |
| Model | **(1) Sex = F** | **(2) Sex = F** | **(3) Sex = M** | **(4) Sex = M** |
| Constant | -1.920 (<0.001***) | -2.003 (<0.001***) | -2.645 (<0.001***) | -2.611(<0.001***) |
| Age (years) | 0.049 (<0.001***) | 0.054 (<0.001***) | 0.063 (<0.001***) | 0.060 (<0.001***) |
| Presence of children (= yes) |  | -0.270 (0.204) |  | 0.134 (0.409) |
|  |  |  |  |  |
| **Observations** | 74 | 74 | 135 | 135 |
| **R²** | 0.160 | 0.179 | 0.307 | 0.311 |
| **Adjusted R²** | 0.149 | 0.156 | 0.302 | 0.300 |
| **Residual Std. Error** | 0.846 (df = 72) | 0.842 (df = 71) | 0.809 (df = 133) | 0.81 (df = 132) |
| **F-Statistic** | 13.74 (df = 1; 72) | 7.754 (df = 2; 71) | 58.91 (df = 1; 133) | 29.73 (df = 2; 132) |
| **F-Test** | RSS: 51.522 | RSS: 50.355/ F: 1.647/ p: 0.204 | RSS: 87.065 | RSS: 86.614/ F: 0.687/ p: 0.409 |

Table G: Multiple linear regression: within-sex analysis of the presence of children. Regression coefficients; p-values in parenthesis. Violation of model assumptions: Residuals were not normally distributed in model 1.
Variables: Dependent variable: “Number of publications (RIN-transformed)”; Independent variables: Age (in years), Sex (reference = female), Presence of children (reference = no). *p < 0.05;**p < 0.01; ***p < 0.001; Coef. = Coefficient; SE = Standard error; M = male; RSS = Residual sum of squares; F = F-value; p = p-value; df = degree of freedom.

| **Presence of children** | **Dependent variable: Number of publications** | | |
| --- | --- | --- | --- |
| Model | **(1)** | **(2)** | **(3)** |
| Constant | -2.535 (<0.001***) | -2.556 (<0.001***) | -2.414 (<0.001***) |
| Age (years) | 0.057 (<0.001***) | 0.058 (<0.001***) | 0.057 (<0.001***) |
| Sex (= M) | 0.316 (0.008**) | 0.328 (0.008**) | 0.128 (0.448) |
| Presence of children (= yes) |  | -0.047 (0.708) | -0.306 (0.120) |
| Sex (= M) x Presence of children (= yes) |  |  | 0.420 (0.088) |
|  |  |  |  |
| **Observations** | 209 | 209 | 209 |
| **R²** | 0.289 | 0.289 | 0.299 |
| **Adjusted R²** | 0.282 | 0.279 | 0.286 |
| **Residual Std. Error** | 0.805 (df = 206) | 0.807 (df = 205) | 0.803 (df = 204) |
| **F Statistic** | 41.825 (df = 2; 206) | 27.814 (df = 3; 205) | 21.793 (df = 4; 204) |
| **F-Test** | RSS: 133.582 | RSS: 133.49/ F: 0.142/ p: 0.706 | RSS: 131.594/ F: 2.94/ p: 0.088 |

Table H: Multiple linear regression: whole cohort; analysis of the presence of children. Regression coefficients; p-values in parenthesis.
Variables: Dependent variable: “Number of publications (RIN-transformed)”; Independent variables: Age (in years), Sex (reference = female), Presence of children (reference = no). *p < 0.05;**p < 0.01; ***p < 0.001; Coef. = Coefficient; SE = Standard error; M = male; RSS = Residual sum of squares; F = F-value; p = p-value; df = degree of freedom.

| **Marital status (reference = single)** | **Dependent variable: Number of publications** | | | |
| --- | --- | --- | --- | --- |
| Model | **(1) Sex = F** | **(2) Sex = F** | **(3) Sex = M** | **(4) Sex = M** |
| Constant | -1.920 (<0.001***) | -2.055 (<0.001***) | -2.645 (<0.001***) | -2.637 (<0.001***) |
| Age (years) | 0.049 (<0.001***) | 0.054 (<0.001***) | 0.063 (<0.001***) | 0.060 (<0.001***) |
| Marital status (= divorced) |  | -1.703 (0.051) |  | 0.215 (0.562) |
| Marital status (= married) |  | -0.088 (0.656) |  | 0.193 (0.289) |
|  |  |  |  |  |
| **Observations** | 74 | 74 | 135 | 135 |
| **R²** | 0.160 | 0.205 | 0.307 | 0.313 |
| **Adjusted R²** | 0.149 | 0.171 | 0.302 | 0.297 |
| **Residual Std. Error** | 0.846 (df = 72) | 0.835 (df = 70) | 0.809 (df = 133) | 0.812 (df = 131) |
| **F-Statistic** | 13.74 (df = 1; 72) | 6.023 (df = 3; 70) | 58.91 (df = 1; 133) | 19.9 (df = 3; 131) |
| **F-Test** | RSS: 51.522 | RSS: 48.766 / F: 1.978/ p: 0.146 | RSS: 87.065 | RSS: 86.297/ F: 0.583/ p: 0.560 |

Table I: Multiple linear regression: within-sex analysis of marital status. Regression coefficients; p-values in parenthesis. Violation of model assumptions: Residuals were not normally distributed in model 1.
Variables: Dependent variable: “Number of publications (RIN-transformed)”; Independent variables: Age (in years), Sex (reference = female), Marital status (reference = single). *p < 0.05;**p < 0.01; ***p < 0.001; Coef. = Coefficient; SE = Standard error; M = male; RSS = Residual sum of squares; F = F-value; p = p-value; df = degree of freedom.

| **Marital status (reference = single)** | **Dependent variable: Number of publications** | | |
| --- | --- | --- | --- |
| Model | **(1)** | **(2)** | **(3)** |
| Constant | -2.535 (<0.001***) | -2.542 (<0.001***) | -2.434 (<0.001***) |
| Age (years) | 0.057 (<0.001***) | 0.057 (<0.001***) | 0.056 (<0.001***) |
| Sex (= M) | 0.316 (0.008**) | 0.313 (0.011*) | 0.116 (0.555) |
| Marital status (= divorced) |  | -0.177 (0.592) | -1.734 (0.035*) |
| Marital status (= married) |  | 0.039 (0.768) | -0.100 (0.595) |
| Sex (= M) x Marital status (= divorced) |  |  | 1.914 (0.032*) |
| Sex (= M) x Marital status (= married) |  |  | 0.268 (0.292) |
|  |  |  |  |
| **Observations** | 209 | 209 | 209 |
| **R²** | 0.289 | 0.291 | 0.308 |
| **Adjusted R²** | 0.282 | 0.277 | 0.288 |
| **Residual Std. Error** | 0.805 (df = 206) | 0.808 (df = 204) | 0.802 (df = 202) |
| **F Statistic** | 41.825 (df = 2; 206) | 20.894 (df = 4; 204) | 15.001 (df = 6; 202) |
| **F-Test** | RSS: 133.582 | RSS: 133.24/ F: 0.266/ p: 0.767 | RSS: 129.931/ F: 2.572/ p: 0.079 |

Table J: Multiple linear regression: whole cohort; analysis of marital status. Regression coefficients; p-values in parenthesis.
Variables: Dependent variable: “Number of publications (RIN-transformed)”; Independent variables: Age (in years), Sex (reference = female), Marital status (reference = single). *p < 0.05;**p < 0.01; ***p < 0.001; Coef. = Coefficient; SE = Standard error; M = male; RSS = Residual sum of squares; F = F-value; p = p-value; df = degree of freedom.

| **R2D:4D** | **Dependent variable: Habilitation** | | | |
| --- | --- | --- | --- | --- |
| Model | **(1)** | **(2)** | **(3)** | **(4)** |
| Constant | -2.955 (<0.001***) | -8.711 (0.083) | -74.587 (0.48) | -91.475 (0.407) |
| Age (years) | 0.067 (0.001**) | 0.068 (<0.001***) | 0.069 (<0.001***) | 0.07 (<0.001***) |
| Sex (= M) | 0.7 (0.021*) | 0.776 (0.013*) | 0.779 (0.013*) | 6.378 (0.553) |
| 2D:4D |  | 5.888 (0.244) | 142.898 (0.515) | 173.904 (0.444) |
| 2D:4D² |  |  | -71.199 (0.532) | -85.264 (0.466) |
| Sex (= M) x 2D:4D |  |  |  | -5.781 (0.602) |
|  |  |  |  |  |
| **Observations** | 209 | 209 | 209 | 209 |
| **Null deviance (df)** | 286.738 (208) | 286.738 (208) | 286.738 (208) | 286.738 (208) |
| **Residual deviance (df)** | 266.02 (206) | 264.648 (205) | 264.263 (204) | 263.99 (203) |
| **Pseudo R² (McFadden)** | 0.072 | 0.077 | 0.078 | 0.079 |
| **Likelihood ratio test** | LL: -133.01 | LL: -132.324/ ChiSq: 1.372/ p: 0.241 | LL: -132.132/ ChiSq: 0.385/ p: 0.535 | LL: -131.995/ ChiSq: 0.274/ p: 0.601 |
| **AIC** | 272.02 | 272.648 | 274.263 | 275.99 |

Table K: Multiple logistic regression: whole cohort; analysis of the right hand digit ratio. Regression coefficients; p-values in parenthesis.

Variables: Dependent variable: “Habilitation”; Independent variables: R2D:4D, R2D:4D², Sex (reference = female), Age (in years).

*p < 0.05;**p < 0.01; ***p < 0.001; OR = odds ratio; CI = confidence interval; M = male; LL = Log-likelihoods; x² = likelihood ratio Chi-squared statistic; p = p-value; df = degree of freedom; AIC = Akaike Information Criterion.

| **L2D:4D** | **Dependent variable: Habilitation** | | | |
| --- | --- | --- | --- | --- |
| Model | **(1)** | **(2)** | **(3)** | **(4)** |
| Constant | -2.955 (<0.001***) | -5.824 (0.242) | -23.847 (0.782) | -6.547 (0.946) |
| Age (years) | 0.067 (0.001**) | 0.068 (<0.001***) | 0.069 (<0.001***) | 0.068 (0.001**) |
| Sex (= M) | 0.7 (0.021*) | 0.74 (0.018*) | 0.736 (0.019*) | -3.477 (0.744) |
| 2D:4D |  | 2.9 (0.559) | 40.254 (0.822) | 7.108 (0.971) |
| 2D:4D² |  |  | -19.342 (0.834) | -3.524 (0.972) |
| Sex (= M) x 2D:4D |  |  |  | 4.351 (0.692) |
|  |  |  |  |  |
| **Observations** | 209 | 209 | 209 | 209 |
| **Null deviance (df)** | 286.738 (208) | 286.738 (208) | 286.738 (208) | 286.738 (208) |
| **Residual deviance (df)** | 266.02 (206) | 265.678 (205) | 265.635 (204) | 265.477 (203) |
| **Pseudo R² (McFadden)** | 0.072 | 0.073 | 0.074 | 0.074 |
| **Likelihood ratio test** | LL: -133.01 | LL: -132.839/ ChiSq: 0.342/ p: 0.558 | LL: -132.817/ ChiSq: 0.043/ p: 0.835 | LL: -132.738/ ChiSq: 0.158/ p: 0.691 |
| **AIC** | 272.02 | 273.678 | 275.635 | 277.477 |

Table L: Multiple logistic regression: whole cohort; analysis of the left hand digit ratio. Regression coefficients; p-values in parenthesis.

Variables: Dependent variable: “Habilitation”; Independent variables: L2D:4D, L2D:4D², Sex (reference = female), Age (in years).

*p < 0.05;**p < 0.01; ***p < 0.001; OR = odds ratio; CI = confidence interval; M = male; LL = Log-likelihoods; x² = likelihood ratio Chi-squared statistic; p = p-value; df = degree of freedom; AIC = Akaike Information Criterion.

| **Dr-l** | **Dependent variable: Habilitation** | | | |
| --- | --- | --- | --- | --- |
| Model | **(1)** | **(2)** | **(3)** | **(4)** |
| Constant | -2.955 (<0.001***) | -2.891 (<0.001***) | -2.837 (0.001**) | -2.828 (0.001**) |
| Age (years) | 0.067 (0.001**) | 0.066 (0.001**) | 0.069 (<0.001***) | 0.068 (0.001**) |
| Sex (= M) | 0.7 (0.021*) | 0.697 (0.022*) | 0.707 (0.022*) | 0.699 (0.024*) |
| Dr-l |  | 5.624 (0.422) | 0.349 (0.964) | 7.497 (0.584) |
| Dr-l² |  |  | -412.541 (0.129) | -403.758 (0.145) |
| Sex (= M) x Dr-l |  |  |  | -10.211 (0.525) |
|  |  |  |  |  |
| **Observations** | 209 | 209 | 209 | 209 |
| **Null deviance (df)** | 286.738 (208) | 286.738 (208) | 286.738 (208) | 286.738 (208) |
| **Residual deviance (df)** | 266.02 (206) | 265.37 (205) | 262.593 (204) | 262.184 (203) |
| **Pseudo R² (McFadden)** | 0.072 | 0.075 | 0.084 | 0.086 |
| **Likelihood ratio test** | LL: -133.01 | LL: -132.685/ ChiSq: 0.65/ p: 0.42 | LL: -131.296/ ChiSq: 2.777/ p: 0.096 | LL: -131.092/ ChiSq: 0.409/ p: 0.522 |
| **AIC** | 272.02 | 273.37 | 272.593 | 274.184 |

Table M: Multiple logistic regression: whole cohort; analysis of the Dr-l. Regression coefficients; p-values in parenthesis.

Variables: Dependent variable: “Habilitation”; Independent variables: Dr-l, Dr-l², Sex (reference = female), Age (in years).

*p < 0.05;**p < 0.01; ***p < 0.001; OR = odds ratio; CI = confidence interval; M = male; LL = Log-likelihoods; x² = likelihood ratio Chi-squared statistic; p = p-value; df = degree of freedom; AIC = Akaike Information Criterion.

| **Presence of children** | **Dependent variable: Number of publications** | | | |
| --- | --- | --- | --- | --- |
| Model | **(1) Sex = F** | **(2) Sex = F** | **(3) Sex = M** | **(4) Sex = M** |
| Constant | -3.065 (0.026*) | -3.330 (0.018*) | -2.120 (0.025*) | -2.221 (0.025*) |
| Age (years) | 0.070 (0.040*) | 0.084 (0.020*) | 0.066 (0.005*) | 0.068 (0.009**) |
| Presence of children (= yes) |  | -0.763 (0.158) |  | -0.069 (0.869) |
|  |  |  |  |  |
| **Observations** | 74 | 74 | 135 | 135 |
| **Null deviance (df)** | 101.230 (73) | 101.230 (73) | 177.97 (134) | 177.97 (134) |
| **Residual deviance (df)** | 96.649 (72) | 94.569 (71) | 169.36 (133) | 169.33 (132) |
| **Pseudo R² (McFadden)** | 0.045 | 0.066 | 0.048 | 0.049 |
| **Likelihood ratio test** | LL: -48.324 | LL: -47.284/ ChiSq: 2.080/ p: 0.149 | -84.681 | LL: -84.667/ ChiSq: 0.027/ p: 0.869 |
| **AIC** | 100.65 | 100.57 | 173.36 | 175.33 |

Table N: Multiple logistic regression: within-sex analysis of presence of children. Regression coefficients; p-values in parenthesis.
Variables: Dependent variable: “Habilitation”; Independent variables: Age (in years), Sex (reference = female), Presence of children (reference = yes). *p < 0.05;**p < 0.01; ***p < 0.001; M = male; LL = Log-likelihoods; x² = likelihood ratio Chi-squared statistic; p = p-value; df = degree of freedom; AIC = Akaike Information Criterion.

| **Presence of children** | **Dependent variable: Habilitation** | | |
| --- | --- | --- | --- |
| Model | **(1)** | **(2)** | **(3)** |
| Constant | -2.955 (<0.001***) | -3.123 (<0.001***) | -2.930 (<0.001***) |
| Age (years) | 0.067 (<0.001***) | 0.075 (<0.001***) | 0.073 (<0.001***) |
| Sex (= M) | 0.7 (0.021*) | 0.792 (0.013*) | 0.504 (0.249) |
| Presence of children (= 1) |  | -0.339 (0.303) | -0.716 (0.168) |
| Sex (= M) x Presence of children (= 1) |  |  | 0.611 (0.341) |
|  |  |  |  |
| **Observations** | 209 | 209 | 209 |
| **Null deviance (df)** | 286.7 (208) | 286.7 (208) | 286.7 (208) |
| **Residual deviance (df)** | 266 (206) | 264.9 (205) | 264 (204) |
| **Pseudo R² (McFadden)** | 0.072 | 0.076 | 0.079 |
| **Likelihood ratio test** | LL: -133.01 | LL: -132.473/ ChiSq: 1.074/ p: 0.3 | LL: -132.018/ ChiSq: 0.91/ p: 0.34 |
| **AIC** | 272.020 | 272.946 | 274.037 |

Table O: Multiple logistic regression: whole cohort; analysis of the presence of children. Regression coefficients; p-values in parenthesis.
Variables: Dependent variable: “Habilitation”; Independent variables: Age (in years), Sex (reference = female), Presence of children (reference = yes). *p < 0.05;**p < 0.01; ***p < 0.001; M = male; LL = Log-likelihoods; x² = likelihood ratio Chi-squared statistic; p = p-value; df = degree of freedom; AIC = Akaike Information Criterion.

| **Marital status (reference = single)** | **Dependent variable: Number of publications** | | | |
| --- | --- | --- | --- | --- |
| Model | **(1) Sex = F** | **(2) Sex = F** | **(3) Sex = M** | **(4) Sex = M** |
| Constant | -3.065 (0.026*) | -3.328 (0.020*) | -2.120 (0.025*) | -2.168 (0.027*) |
| Age (years) | 0.070 (0.040*) | 0.088 (0.015*) | 0.066 (0.005**) | 0.058 (0.021*) |
| Marital status (= divorced) |  | -16.643 (0.991) |  | 0.317 (0.746) |
| Marital status (= married) |  | -0.865 (0.092) |  | 0.378 (0.408) |
|  |  |  |  |  |
| **Observations** | 74 | 74 | 135 | 135 |
| **Null deviance (df)** | 101.230 (73) | 101.230 (73) | 177.97 (134) | 177.97 (134) |
| **Residual deviance (df)** | 96.649 (72) | 91.766 (70) | 169.36 (133) | 168.68 (131) |
| **Pseudo R² (McFadden)** | 0.045 | 0.093 | 0.048 | 0.052 |
| **Likelihood ratio test** | LL: -48.324 | LL: -45.883/ ChiSq: 4.883/ p: 0.087 | LL: -84.681 | LL: -84.339/ ChiSq: 0.684/ p: 0.711 |
| **AIC** | 100.65 | 99.766 | 173.36 | 176.68 |

Table P: Multiple logistic regression: within-sex analysis of marital status. Regression coefficients; p-values in parenthesis.
Variables: Dependent variable: “Habilitation”; Independent variables: Age (in years), Sex (reference = female), Marital status (reference = single). *p < 0.05;**p < 0.01; ***p < 0.001; M = male; LL = Log-likelihoods; x² = likelihood ratio Chi-squared statistic; p = p-value; df = degree of freedom; AIC = Akaike Information Criterion.

| **Marital status (reference = single)** | **Dependent variable: Habilitation** | | |
| --- | --- | --- | --- |
| Model | **(1)** | **(2)** | **(3)** |
| Constant | -2.955 (<0.001***) | -3.017 (<0.001***) | -2.582 (0.003**) |
| Age (years) | 0.067 (<0.001***) | 0.072 (<0.001***) | 0.069 (<0.001***) |
| Sex (= M) | 0.7 (0.021*) | 0.758 (0.016*) | 0.046 (0.927) |
| Marital status (= divorced) |  | -0.544 (0.525) | -15.414 (0.986) |
| Marital status (= married) |  | -0.200 (0.553) | -0.805 (0.105) |
| Sex (= M) x Marital status (= divorced) |  |  | 15.665 (0.986) |
| Sex (= M) x Marital status (= married) |  |  | 1.118 (0.089) |
|  |  |  |  |
| **Observations** | 209 | 209 | 209 |
| **Null deviance (df)** | 286.7 (208) | 286.7 (208) | 286.7 (208) |
| **Residual deviance (df)** | 266 (206) | 265.4 (204) | 260.9 (202) |
| **Pseudo R² (McFadden)** | 0.072 | 0.074 | 0.09 |
| **Likelihood ratio test** | LL: -133.01 | LL: -132.719/ ChiSq: 0.582/ p: 0.748 | LL: -130.451/ ChiSq: 4.536/ p: 0.104 |
| **AIC** | 272.020 | 275.438 | 274.902 |

Table Q: Multiple logistic regression: whole cohort; analysis of marital status. Regression coefficients; p-values in parenthesis.
Variables: Dependent variable: “Habilitation”; Independent variables: Age (in years), Sex (reference = female), Marital status (reference = single). *p < 0.05;**p < 0.01; ***p < 0.001; M = male; LL = Log-likelihoods; x² = likelihood ratio Chi-squared statistic; p = p-value; df = degree of freedom; AIC = Akaike Information Criterion.
